# Affect and Cognitive Closure in Students—A Step to Personalised Education of Clinical Assessment in Psychology with the Use of Simulated and Virtual Patients

**DOI:** 10.3390/healthcare10061076

**Published:** 2022-06-09

**Authors:** Maciej Walkiewicz, Bartosz Zalewski, Mateusz Guziak

**Affiliations:** 1 Department of Psychology, Medical University of Gdańsk, 80-210 Gdansk, Poland; 2 Department of Psychology of Individual Differences, Diagnosis and Psychometric Research, SWPS University of Social Sciences and Humanities, 03-815 Warsaw, Poland; bartosz.zalewski@swps.edu.pl; 3 Faculty of Medicine, Medical University of Gdańsk, 80-210 Gdansk, Poland; mateusz.guziak@gumed.edu.pl

**Keywords:** affect, interview, clinical reasoning, clinical competence, diagnosis, simulation training, patient simulation, psychology, clinical psychology, students, education, teaching

## Abstract

Introduction: Since there was no general model of competencies to determine a successful clinical assessment, we based our study on the many skills that are needed to perform one. We analysed students’ learning performance based on inner determinants, such as affect and cognitive closure, with the use of two teaching methods (i.e., simulated patient (SP) or virtual patient (VP)). Methods: The sample comprised 56 fifth-year clinical psychology students. The need for closure (NFC) and efficacy in fulfilling the need for closure (EFNC) were measured using standardised questionnaires. The authors’ VP and SP tools were used to teach and measure the effectiveness of learning psychological interview techniques and clinical reasoning. Clinical interview skills included building contact with the patient, gathering important information and making mistakes. Clinical reasoning skills were divided into eight dimensions for the assessment of mental health. Results: Affect and cognitive closure are important psychological variables in anticipating and developing interview and clinical reasoning skills for psychology students. The simulated patient was more effective for interview skills, while the virtual patient was a beneficial teaching tool for most clinical reasoning skills. Virtual patient training was a useful teaching method for students with a low EFNC, probably because it provided a stable and strong structure. Simulated patient training was effective for people with a high EFNC, presumably because it allowed them to build on their advanced structuring skills. Conclusions: Affect and cognitive closure can be used to identify students’ learning abilities to provide a more personalised education. The results of the present study may be useful for evaluating different teaching methods, monitoring their effectiveness and enhancing students’ performance.

## 1. Introduction

Questions concerning the inner determinants of the acquisition of diagnostic skills are surprisingly lacking in publications on the psychological profession [1]. Traditional training in clinical diagnosis stresses the need to build a cooperative relationship with the patient, collect data [2,3] and analyse them to integrate complex information and social interactions involving social exposition and emotional burden [4]. 

To conduct a successful diagnostic interview in psychology, students must learn to regulate their emotions and recognise those of others. Therefore, *affect*—as part of more advanced states, such as emotions, feelings, preferences, moods and traits [5,6]—plays an important role in learning [7,8,9]. A small, temporary change in affective state can significantly impact the performance of a challenging cognitive task requiring flexible thought or a clear solution [10]. Affect is usually classified as either positive (PA) and negative (NA) [11]. The former facilitates higher problem-solving and clinical reasoning abilities [12], while the latter modulates cognitive functions. Positive affect is known to decrease cognitive inhibition in response to emotionally marked material [13]. In learning clinical skills, higher PA can improve verbal fluency [14,15] and reduce interference between competing responses, which results in faster reaction time between congruent and incongruent stimuli [16].

The cognitive–motivational aspects of reasoning are expressed through the need for cognitive closure (NFC) and efficacy in fulfilling the need for closure (EFNC) [17]. The NFC is defined as the desire for an answer to a given problem [18]. In the context of clinical reasoning in psychology, a high NFC is associated with: a more limited search for information before a decision is made [19]; higher ratings of confidence after a decision is made [20]; a stronger preference for familiar choices instead of new options; and a tendency to stereotype [21,22,23]. Efficacy in fulfilling the need for closure refers to the individual’s ability to reach swift decisions and create structure in life. The level of EFNC is determined by the extent to which individuals can use different styles of information processing according to their NFC [24].

Since there was no general model of competencies to determine a successful clinical assessment, we based our study on the skills that are known to be required: clinical interview and reasoning skills.

*Clinical interview skills* involve building contact with the patient, gathering important information and making mistakes [25]. *Clinical reasoning skills* refer to: (1) The ability to recognise patients’ negative and positive aspects of functioning [26]; (2) The reactance of a patient. A reactant patient is easily provoked and responds oppositional to perceived external demands [27]; (3) The coping style of a patient, which is an enduring personality trait when a person confronts new problematic situations. We distinguish two styles: externalising (impulsive, stimulation-seeking, extraverted) and internalising (self-critical, inhibited, introverted) [28]; (4) The stage of change of a patient, which represents a person’s readiness for psychological change, defined as a period of time and set of tasks needed for movement to the next stage [28]; (5) The cognitive errors in clinical reasoning: (a) Confirmation bias: the tendency to look for confirming evidence to support a diagnosis rather than look for disconfirming evidence to refute it [29]. (b) Overconfidence bias: a universal tendency to believe we know more than we do [29]. (c) Multiple alternative bias: a multiplicity of options on a differential diagnosis may lead to significant conflict and uncertainty [29]. (d) Overpathologisation bias: not explicitly mentioned in medical literature, however, very similar to other biases distinguished, e.g.,: premature closure, representativeness restraint, search satisficing [30], ascertainment bias, diagnosis momentum [29] and focusing effect [31]; (6) Adequacy of collected data refers to the quality and completeness of collected diagnostic data presented by the SP and VP; (7) The general quality of assessment, which refers to the general measurement of quality of diagnosis.

We applied two teaching methods to investigate a wide range of diagnostic competencies. One involved direct contact, while the other was computer mediated; this enabled the measurement of dependent variables at two levels of interaction. The first method—simulated patient (SP)—demands significant involvement, since the learner has to perform a complex diagnostic activity, bridge cognitive biases, formulate diagnostic interventions and more [32,33]. The second—virtual patient (VP)—entails a lower cognitive–emotional burden because the student does not have direct contact with the patient. At the same time, they can be provided with information on the steps they have to take [34].

The present study complements the literature by assessing the value of SP and VP in the acquisition of both interview and clinical reasoning skills. The goal of the study was to analyse students’ learning performance based on affect and cognitive closure with the use of the simulated patient (SP) and virtual patient (VP).

## 2. Materials and Methods

**Material**. The sample comprised 56 fifth-year clinical psychology students (86% female) at the University of Social Sciences and Humanities, Warsaw, Poland (average age *M* = 28.48; *Me* = 24; *SD* = 8.53). Participation in the study was one of the options in a mandatory student internship. Respondents could withdraw from participation at any time. In total, 69 students applied for the project: SP N = 38; VP N = 31. N = 56 took part in the study after the two-day training course. SP initial session N = 25; SP final session N = 24. VP initial session N = 31; VP final session N = 27. The number of participants who completed the entire project from the beginning to the end was N = 51.

**Figure 1:**
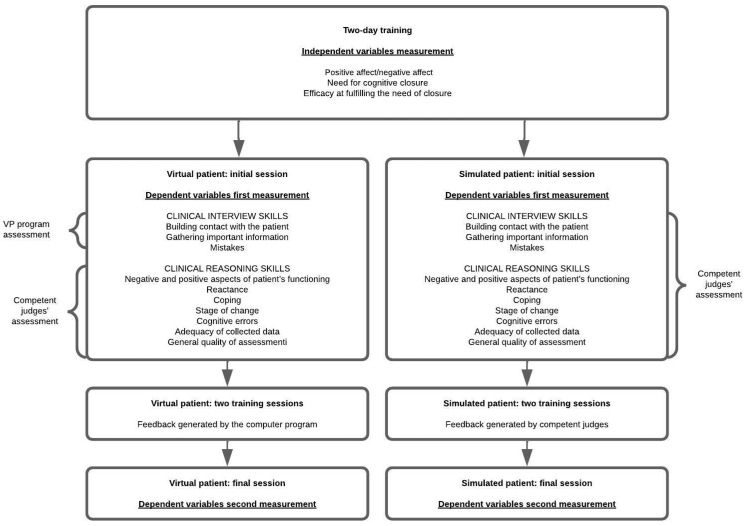
The study procedure.

**Procedure**. The study was carried out between September 2017 and May 2018. The participants took part in a two-day training course (12 h) on the process of clinical diagnosis. The independent variables NFC and EFNC were measured. Four SP sessions and four VP sessions were conducted. Half of the randomly selected participants took part in SP sessions, and the other half took part in VP sessions. Each session consisted of completing the SP or VP procedure and writing a clinical diagnosis. The goal of the initial sessions was to assess the primary level of students’ *diagnostic skills* (dependent variables). For the SP sessions, the level of clinical interview and reasoning skills were assessed by competent judges, as were the participants’ diagnoses of the patients. For the VP sessions, the level of clinical interview skills was assessed by the VP computer program, while the participants’ clinical reasoning skills were assessed by competent judges. The second and third sessions were used for training. The SP participants received feedback from a competent judge who observed the interview, and the VP students received feedback generated by the VP program. The final level of the participants’ diagnostic skills (dependent variables) was assessed during the final sessions (see Figure 1).

**The independent variables** were measured using two questionnaires. Affect was measured using the Positive and Negative Affect Schedule-Trait (PANAS) [11,35,36]. This questionnaire contains 30 items on two subscales that assess a person’s positive affective states (i.e., active, alert, attentive, determined, enthusiastic, excited, inspired, interested, proud and strong) and negative ones (i.e., afraid, ashamed, distressed, guilty, hostile, irritated, nervous, scared and upset) [37,38,39,40]. The PANAS uses a Likert-type scale, with answers ranging from *very slightly* or *not at all* (1) to *extremely* or *very much* (5).

To measure NFC, we used Webster and Kruglanski’s (1994) NFC scale. Respondents rated the 32 items on a 6-point scale (from 1 = *completely disagree* to 6 = *completely agree*). This scale has good psychometric properties [41].

Efficacy at fulfilling the need for closure was measured using nine items (high and low levels) and a 6-point scale (from 1 = *completely disagree* to 6 = *completely agree*). A higher score implied a higher level of EFNC. This scale has good psychometric properties [24].

**The dependent variables**:

*Clinical interview skills.* These were measured using the VP authors’ computer tool, which consists of short recordings of a person playing the role of the patient in a diagnostic psychological consultation lasting 20–25 min. The recordings were based on case studies of real patients with severe mental disorders using the Keyes and Lopez dimension model, which is based on a description of positive and negative aspects of the patient’s functioning, resulting in four types of profiles, from healthy persons to patients with more severe mental disorders [42,43]. Each prototype was described for both female and male roles. In Appendix A, we provide examples of the individual patients played. Each prototype included a description of the problems reported by the patient, the duration and severity of symptoms, a short history of the patient’s personal and professional life and the environment from which s/he came from, including a history of romantic relationships and relations with loved ones. Finally, a description of the behaviour during the interview and the objectives of the relevant therapy were added. The type of disorder was described by indicating the dominant symptoms, the nosological diagnosis of the disorder, the level of reactance and readiness for change and the style of coping. In the ‘healthy’ profile, there was no individual disorder or extensive symptoms; the simulant talked about her/his greatest concerns, being significantly disturbed by the behaviour of a loved one. The participants’ task was to recognize that this particular SP does not manifest disorders but talks about another person’s mental difficulties.

The participants watched nine successive fragments of a patient interview, and after each one, they chose one of two diagnostic interventions. The program enables 512 combinations of selection paths arranged in a decision tree. In the second and third VP training sessions, the participants received feedback after each decision (Appendix B).

For the SP, interview skills were measured live by competent judges on scales based on: (1) building contact with the patient; (2) gathering important information; and (3) making mistakes. The SPs session lasted 50 min, which is the standard time for a psychological interview. The simulated patients were experienced academic teachers and practising clinicians (i.e., not actors). They developed their roles during 10 h workshops and played the same ones with all participants (Appendix A).

*Clinical reasoning skills* were measured by a tool (designed by the authors) that estimates the way one formulates diagnostic hypotheses after the interview with an SP or a VP (Appendix A). The competent judges evaluated whether students applied the following dimensions of patient characteristics in their clinical reasoning: (a) *negative and positive aspects of a patient’s functioning*; (b) *reactance*; (c) *coping style*; (d) *stage of change*; (e) *cognitive errors*; (f) *adequacy of collected data*; and (g) the *quality of assessment* (Appendix D, Appendix E, Appendix F). The competent judges were experienced, academic teachers. They and the participants were anonymous.

**Statistical analysis**. IBM SPSS 25 software was used for data analysis. Pearson correlations were conducted to investigate the relationships in the data.

**Ethics statement**. The present study was conducted in accordance with the guidelines of the Ethical Review Board at the University of Social Sciences and Humanities in Warsaw, Poland, and was reviewed and approved (decision 23/IV/11-12). Written consent was obtained from all participants.

## 3. Results

*Interview skills*

*Building contact with the patient* presents: a low negative correlation with the *PA* in the final VP session, a low negative correlation with *EFNC* in the initial SP and the final VP session.

*Mistakes*presents a low negative correlation with *EFNC* in the initial SP session.

*Clinical reasoning skills*

*Negative aspects of patient’s functioning* presents a low positive correlation with *EFNC* in the initial VP session.

*Positive aspects of patient’s functioning* presents a low negative correlation with *NFC* in the final VP session.

*Reactance* presents a negligible positive correlation with *PA* in the final SP session.

*Coping style* presents a low positive correlation with *PA* in the final VP session.

*Confirmation bias* presents a low positive correlation with *PA* in the initial VP session, a low positive correlation with *EFNC* in the initial VP session.

*Overconfidence bias* presents a negligible positive correlation with *EFNC* in the initial SP session.

*Multiple alternative bias* presents a low negative correlation with *PA* in the initial SP session, a negligible positive correlation with *NFC* in the final SP session.

*Overpathologisation bias* presents a negligible positive correlation with *PA* in the initial VP session, a low positive correlation with *NFC* in the initial SP, a low positive correlation with *NFC* in the final VP, a low positive correlation with *EFNC* in the final SP, a low positive correlation with *EFNC* in the initial VP session, a moderate positive correlation with *EFNC* in the final VP session.

*Adequacy of collected data* presents a low positive correlation with *PA* in the initial VP, a negligible positive correlation with *EFNC* in the initial VP session (see Table 1).

## 4. Discussion

We analysed students’ learning performance based on inner determinants, such as affect and *cognitive closure*, with the use of two teaching methods (i.e., simulated patient (SP) or virtual patient (VP)). The results showed that SP was more effective for all interview skill variables and VP for most clinical reasoning skill variables.

In the SP task, participants with high PA and EFNC were experiencing a significant amount of stress. A high level of PA creates a natural tendency to regulate tension through positive emotions and thus satisfaction at having completed a task. The need to *build contact* is neglected as a result. The highly structured VP program did not allow the participants to generate their own responses. Therefore, it may be detrimental for people with a high EFNC because they build structure naturally on their own [24].

The SP training provided expressive feedback and immediate responses to participants’ errors, so that high-EFNC participants made fewer *mistakes*. Participants with a low EFNC could not structure the interview with the SP and, therefore, made more and more *mistakes* over time. Those *mistakes* were probably based on a momentary but illusory sense of confidence and resulted in the participants offering incorrect advice and misinterpreting the patient’s behaviour.

The *need for cognitive closure* was not associated with any interviewing skill for either SP or VP because it involves cognitive processes (clinical reasoning) rather than information gathering (interviewing). Another possible explanation is that the interview framework (contact/structure/mistakes) fulfils the NFC, and the participants conducted the interview regardless of their NFC level, implying that it does not inhibit interview learning.

*Clinical reasoning skills* appeared to be much more complex variables and generated more results. Participants had to analyse patient responses in several dimensions to formulate key diagnostic hypotheses.

During SP, higher NA levels were associated with less effective diagnoses of the patient’s *reactance*, perhaps because the participants were more self-centred, mood regulating and lacking the flexibility they needed to recognise that the patient may have had internal reasons for resistance or that the diagnostician was building an unfavourable therapeutic alliance, which is consistent with the *reactance* definition [27].

During VP training, participants with higher NFC may have narrowed their processing of psychopathology and formed their hypotheses adequately. They may also have realised they should choose the most significant data and quickly abandoned processing other information that may have led to clear, unambiguous conclusions.

Identifying *coping style* is a relatively simple diagnostic skill because different ones have clear indicators. Participants with higher PA were better able to recognise them.

*Stage of change* may not have been noticed or considered as a significant source of information because it does not appear in any dimension to describe the patient’s characteristics. We supposed that because our respondents were at a very early stage of their education; they had not paid any attention to it. Perhaps they believed that patients seeking psychological counselling would be motivated to change. Experienced doctors know that patients’ motives can be more complex; indeed, people experiencing emotional distress are often ambivalent about seeking help at all.

The majority of *cognitive errors* were eliminated with our training. This is a promising result because it suggests that both SP and VP tools are effective in reducing mistakes and strengthening critical reasoning skills.

High-EFNC participants stopped committing *confirmation bias*, which suggests that they reduced their cognitive distortions through the training. The VP participants recognised more and more alternative interpretations of the decision paths and made more and more composite diagnoses throughout their training. This effect did not occur in SP because it allowed participants to collect complex and multi-layered data immediately.

High-EFNC participants learned how to assess more complex structures and the multidimensional nature of the patient in SP reducing *overconfidence bias*. They also reduced their *multiple alternative biases*. This meant that they built more complex and multifaceted hypotheses after training. On the other hand, those with a low NFC built too many alternatives and too many hypotheses and were unable to verify them with any reliability.

*Overpathologisation bias* refers to an excessive focus on the negative aspects of a patient’s functioning [31]. The participants who focused overly on learning how to diagnose psychopathology during training likely gave too much attention to this aspect. Because it is a highly structured tool, the VP program seemed not to help students with a high NFC, while a less structured approach (i.e., SP) did. In theory, we would expect the opposite for a complex task such as *clinical reasoning* [24]. Participants with a high EFNC in the SP group began to pathologise patients excessively. They quickly found a clear structure for organising diagnostic information and did not allow other alternative explanations. A high level of EFNC did not support participants in building complex, multifaceted diagnoses. The opposite was the case for participants with a low EFNC who perhaps need more time to structure information and generate hypotheses. Their low synthesising skills allow them to build hypotheses from a smaller but they are more diverse amount of data, covering both positive and negative aspects of functioning.

Participants with higher PA in the VP group were more able to assess the information at the beginning of training but stopped thereafter, perhaps because they became more critical. Moreover, the previously described mechanism seems to be confirmed by the results obtained from the emotional burden scales.

The VP tool provided only a limited amount of information about the patient, but the participants in the SP group could ask questions freely and obtain more data as a result. They were then able to propose hypotheses based on a wider range of information than was the case with the VP.

## 5. Conclusions

Affect and cognitive closure are important psychological variables in anticipating and developing interview and clinical reasoning skills among psychology students. SP training seems to be more effective for all interview skills, whereas VP training can be a beneficial teaching tool for most clinal reasoning skills. VP training is a useful teaching method for students with a low EFNC, probably because it provides a stable and strong structure. SP is effective for people with a high EFNC, presumably because it allows them to build on their high structuring skills. Both affect and cognitive closure might help in identifying students’ learning abilities and matching their competencies to provide a more personalised education.

### Limitations

The study was conducted at one university and involved fifth-year clinical psychology students, limiting its applicability to the general population of a trainee. All results were based on interviews and clinical reasoning conducted with simulated and virtual patients. Changes in emotional distress appear to be amenable to investigation not only by self-report but also by physiological measures, but this was not the case in the present study.

## Figures and Tables

**Table 1:** Relationships between affect, need and efficacy at fulfilling the need for closure of diagnosticians and psychological assessment skills learnt through the use of simulated and virtual patients.

	Positive Affect—PA (PANAS)	Negative Affect—NA (PANAS)	Need for Cognitive Closure (NFC)	Efficacy at Fulfilling the Need for Closure (EFNC)
**Interview Skills**				
1. Building contact with the patient	VP/F r(32) = −0.421; *p* = 0.016			SP/I r(56) = −0.337; *p* = 0.011 VP/F r(32) = −0.485; *p* = 0.005
2. Gathering important information				
3. Mistakes				SP/I r(47) = −0.315; *p* = 0.031
**Clinical Reasoning Skills**				
1.1. Negative aspects of patient’s functioning				VP/I r(41) = 0.314; *p* = 0.046
1.2. Positive aspects of patient’s functioning			VP/F r(43) = −0.390; *p* = 0.010	
2. Reactance		SP/F r(47) = 0.291; *p* = 0.047		
3. Coping style	VP/F r(42) = 0.315; *p* = 0.042			
4. Stage of change				
5.1. Confirmation bias	VP/I r(48) = 0.310; *p* = 0.032			VP/I r(41) = 0.441; *p* = 0.004
5.2. Overconfidence bias				SP/I r(55) = 0.290; *p* = 0.031
5.3. Multiple alternative bias	SP/I r(51) = −0.303; *p* = 0.031		SP/F r(48) = 0.289; *p* = 0.046	
5.4. Overpathologisation bias	VP/I r(48) = 0.298; *p* = 0.040		SP/I r(46) = 0.308; *p* = 0.037 VP/F r(43) = 0.343; *p* = 0.024	SP/F r(49) = 0.305; *p* = 0.033 VP/I r(28) = 0.395; *p* = 0.038 VP/F r(21) = 0.556; *p* = 0.009
6. Adequacy of collected data	VP/I r(47) = 0.306; *p* = 0.036			VP/I r(51) = 0.298; *p* = 0.034
7. Quality of assessment				

SP—simulated patient; VP—virtual patient; I—initial session; F—final session.

## Data Availability

The data presented in this study are available on request from the corresponding author.

## Appendix A

**Example SP and VP profiles**

In Appendix A, we provide examples of the individual patients played with the full description of the problems reported by the patient, the duration and severity of symptoms, a short history of the patient’s personal and professional life and the environment from which s/he came from, including a history of romantic relationships.

**Profile 1, Miss X, 33**

The patient visits the therapy centre because she would like to take better care of herself. She works as a choreographer. She is highly valued for her competence but still only holds the position of assistant, not the main production choreographer. She dreams of being noticed and discovered but thinks that there are many jealous, unkind women in her environment who compete unfairly. The patient would not like to compete in the way they do; she values good relations and does not want to stand out at the expense of others. At the same time, she highly values her professional competence. She would like to be able to effectively strive for a position that would reflect her competencies; at the same time, she would like to be able to do it her own way—without entering into the unhealthy competition.

Currently, she is in a relationship with her partner, D., who is a wealthy businessman. They live in a house near the capital city of (COUNTRY) with a beautiful garden, a fountain and their own conservatory. However, being there, away from people and the city, she declares that she works at her best among people. She organises her social life for herself and her partner. She describes her partner as cool, slightly withdrawn, showing feelings in a very reserved way.

The patient is emotionally in a very difficult situation; as she explained, tired of the relationship with a cool partner, for the first time in her life, she became involved in an affair with her colleague, M. He is brilliant—very talented, sociable, affectionate. The patient feels that he is her ‘soulmate’—a man with a passion for life, with interests that are close to hers; he is like a male version of herself. The patient feels that she has been waiting for someone like that all her life. At the same time, she does not feel strong enough to end her relationship with her partner. She told him about the affair; she wants to be honest in her relationship. D. was angry, but he decided to forgive her and strive to work at their relationship. He says that the patient is the most important person in his life.

The patient is trapped; she loves M. but does not want to be disloyal to D. The patient’s mother advises her to end the affair, commenting that the relationship with M. is yet another relationship in the patient’s life, and it may be that after 2 or 3 years, she will become bored with her relationship with M. She is already 33 years old, and it would be good to stay in a stable relationship. The patient acknowledges her mother is partly right, but she does not want to duplicate her mother’s own story; her mother is in an emotionally distanced relationship with her father. The father is highly placed in a largely state-owned company; he earns a lot; he is often not at home. He always emphasised that the patient must not bring shame on him. The patient feels very sorry for her mother about this relationship; she understands that her mother had to look for warmth in relationships outside of marriage. However, she would like to be in a relationship with someone she loves and who shares her passions and energy for life and knows how to show her commitment.

Example of a situation (work)

Recently, the patient was employed in the production of a short film. She was very pleased with the results of her work, but the director did not praise her. The production budget was large; it was clear nothing was saved. The patient was employed by the head of the choreography. After implementation, the boss delayed the transfer of payment for a long time; it was frustrating. The patient later learned that she earned a fraction of what the boss did, although she did almost nothing; all the best ideas were the patient’s ideas. This situation aroused anger, indignation and powerlessness; the patient would not dare to apply for an independent position, going against the boss who had previously hired her.

Example of a situation (relations)

The patient came to (CITY) last week because of the promotion of the film on which she collaborated. Her partner, D., forbade her to participate in the informal celebration that took place after the official event. Her friends were very surprised that she was leaving, and they started calling her. In the end, she gave in and joined them. M.—with whom she had an affair—was also there. They went for a walk together, talked for a long time; it was amazing, as if they had known each other for many years. Later, however, the patient decided that she was behaving badly; she barely said goodbye and went to the friend she was supposed to be sleeping over with. Her friend was surprised that she came without M. and suggested that they go over to M.’s together. The patient arrived, and then, after several minutes, the friend said goodbye. The patient finally spent the night with M.; as she states, it was incredibly close and significant, but she felt she was being unfair toward D.

Expectations for the therapy

Get a job as the first choreographer, be able to take care of her needs in her personal life, be good at making choices in a way that does not hurt others.

**Profile 2, Miss Y, 26**

She is in a relationship, but she is not able to start having sexual life with her partner. She is also very tired in her work; she works in a clothing store in a large shopping centre.

She is highly valued at work, but she works over 12 h a day; she does not have free weekends, she often works at unfavourable hours, and she is stating that the boss is harassing her. She is afraid of getting angry with the boss and does not refuse him. She is afraid that she will not find a better job. She does not feel confident enough to send a CV to another place.

She studies journalism; she is doing great, she has a scholarship and a lot of support from the faculty authorities (lecturers appreciate her commitment and competence). She has a large group of friends and two close ones. Together, they go on vacation to the lake district in (COUNTRY). They like to read, talk and spend time together.

Now, she lives with her boyfriend. Decorating their new place gave them a lot of joy. They live with the patient’s boyfriend’s father, who often goes away and does not interfere in their affairs; she and her partner often have the whole apartment to themselves. Recently, they adopted a cat whom they called Harold. The client values contact with the animal very much.

Currently, she is afraid that her lack of ability to have intercourse will harm their relationship. She is afraid that her boyfriend will leave her, although he declares that this is not true. However, she thinks he cannot stand it any longer. The patient is afraid that a relationship without an element of intercourse is so unsatisfactory for a man that she expects that, if she does not feel able to have sex with her boyfriend soon, he will start cheating on her or will leave her. The partner finances her psychologist consultation and wants to finance her therapy because she does not earn very much. The patient would like to be able to have the courage to change jobs. She would like to earn more, so as not to be a burden on her boyfriend; she wants him to be able to afford nice clothes and enjoy himself.

When she thinks about changing jobs, she is afraid that people reading her CV will laugh at her lack of experience. She is afraid that even if she finds another job, she will not be better at it than at her current job, that she will be a victim of harassment again. She feels an irrational fear of men who remind her of a man who had molested her in the past. It was a father’s friend; the patient does not want to talk about this anymore. She is afraid that she might meet someone similar to this man at an interview or a new job. She knows that it would paralyse her; she would not be able to speak, and she could not work.

She would like to undergo therapy focused on dealing with the fear of intercourse and the fear of looking for a new job. She emphasises that she does not want to work with a sexologist because she thinks her problems are emotional, not physical.

An example of a situation in which the patient was afraid of her boyfriend leaving her

Recently, when she was with her partner in a shopping centre, he noticed a dress in the shop and asked her to try it on. The patient put on the dress and could see that she looked attractive in it. The boyfriend said that she looked great. However, she thought that she was now attracted to him, and she felt horror—a tightness in the stomach and tension in the arms and neck. She thought that he would like to have sex with her, and she was very afraid of it; she really does not want to have sex. She thought her reaction was abnormal; she was taught that women like to please their partners. She felt guilty, strange. She thought her boyfriend would not be able to tolerate it in the long run. She told him that she did not like this dress and immediately hung it up. The whole evening was tense and sad; she wanted to cry. At the same time, she tried to hide it from her boyfriend.

An example of a situation in which the patient was afraid to look for a new job.

Yesterday, the patient looked at job offers in the newspaper. She began to check if she had the skills required for several positions that seemed attractive to her. First, she decided that she actually had these skills, and then, she imagined that she would be asked about them during the interview (e.g., the interview would be in English). She imagined how she was losing the thread in the interview, and the interviewer comments on her lack of skills and accuses her of lying; she had written that she knew English. The patient feels fear paralysing her; she pushes the newspaper away and decides not to think about it anymore.

## Appendix B. The Diagnostic Hypothesis Written by the Participant after SP and VP

How do you assess the importance of diagnostic data obtained by you in the interview?	I have enough information to design adequate feedback
	After completing certain data, I could design adequate feedback
	Most of the information needed to provide adequate feedback is missing
List the individual diagnostically relevant information you have collected and how to interpret it in relation to the diagnostic question	
………………………………………………………………………………………………………………… ………………………………………………………………………………………………………………… …………………………………………………………………………………………………………………	

Now, we ask you to evaluate your level of confidence in your hypotheses. Each subsequent hypothesis has a corresponding scale. Rate the confidence level of each hypothesis on a scale from 1 (very low confidence) to 10 (very high confidence) by ticking the appropriate box on each scale.

Below are 10 scales. If the number of hypotheses you have formulated is less than 10, use as many scales as you have formulated/formulated hypotheses, and leave the other scales blank.

How would you formulate the project of recommendation at this stage of work? If you would like additional data to formulate adequate recommendations—specify what information you lack and how you could obtain it.
………………………………………………………………………………………………………………… …………………………………………………………………………………………………………………
On what did you base your project of recommendation and, if applicable, a plan for obtaining further information (indicate what variables you took into account and why)?
………………………………………………………………………………………………………………… ………………………………………………………………………………………………………………… ………………………………………………………………………………………………………………… …………………………………………………………………………………………………………………

Determine your confidence in the recommendations you have made by ticking the appropriate box on each scale.

Below are 10 scales. If the number of hypotheses you have formulated is less than 10, use as many scales as you have formulated/formulated hypotheses, and leave the other scales blank.

## Appendix C. Scheme for Competent Judges to Give Feedback to the Diagnosticians after SP

The competent judge comprehensively observed the interview with the SP, made notes and finally gave feedback to the diagnostician.

1. To what extent did the participant build contact with the patient (e.g., using minimal verbal reactions, paraphrases, clarifications, a reflection of emotions)?

Insufficiently/partially/fully (correct).

Can you provide (some) examples?:

2. Did the diagnostician make mistakes in building contact (e.g., interpreting, suggesting, evaluating, advising)?

If so—can you provide (some) examples?:

3. To what extent did the diagnostician use the structure of the interview (examining areas such as the problem, an example of a problem situation, self-diagnosis, motivation and expectations, areas of well-being)?

Insufficiently/partially/fully (correct).

Can you provide (some) examples?:

4. Did the diagnostician make mistakes in following the structure (e.g., omitting specific areas worth exploring or not asking/deepening after receiving an incomplete/superficial answer)?

If so—can you provide (some) examples?:

5. The general rule when conducting an interview is to alternate between building contact with patients and following the structure (with the primacy of building contact). To what extent did the diagnostician comply with this rule?

Insufficiently/partially/fully (correct).

Can you provide (some) examples?:

6. Did the diagnostician make mistakes in the alternation between building contact with the patient and structure (e.g., mainly followed the patient, trying to build contact, or mainly ‘questioned’, guided by the structure)?

If so—can you provide (some) examples?:

7. Diagnostician’s reactions/questions/comments.

**Feedback example for competent judges to give feedback to the diagnosticians after SP**

During the interview, you built proper contact with the patient. You used the patient’s wording and reflected his/her emotions. For example, when you noticed that decorating his/her own home was exciting for the patient. Thanks to this, the patient could feel listened to and understood, which probably had a positive effect on his/her readiness to disclose information about herself.

At one point in the interview, you made a mistake in assessing the patient’s behaviour. Admittedly, it was a positive assessment; you told his/her that it is very good that s/he wants to develop his/her passion. However, even a positive assessment is a mistake because it is putting yourself in a superior position to the patient—in the position of a person authorized to review his/her behaviour. I imagine that at that point in the interview, you might have been guided to highlight an area that is important to the patient and which concerns his/her resources and well-being; is that a correct guess? In such a situation, it is worth exploring the topic using, for example, a paraphrase or reflection of emotions (‘I hear that this plan is something very important to you; you are happy about your brand of accessories, right?’).

During the interview, you followed the structure; you collected important information by asking about the problem and carefully examining an example of a problem situation. The moment when you asked about the thoughts accompanying your patient in the discussed example situation seemed especially valuable to me because you obtained a lot of diagnostically important information. However, what was not found out was the question of the patient’s expectations. I understand that there was not enough time to research this area. Nevertheless, it is very important to formulate a recommendation proposal for the patient. The general rule of a clinical interview is to alternate between building contact with the patient and following structure (with greater importance placed on contact). During the interview, you primarily built contact, which is important for the quality of the data collected in the interview. You also asked about individual areas included in the structure of the interview, but there was not enough time to discuss the important issue of the patient’s expectations. Therefore, it is worth focusing on following the structure of the interview in a way that would not disturb building contact. For example, you could use mini-contracts more often, such as: ‘I have the impression that we have touched on an important topic now. However, we will not be able to exhaust this topic during today’s meeting. There are a few other points that seem very important to me for understanding your situation. So, can we close this topic for now and move on to the next one?’

To sum up, during the interview, you built your alliance with the patient very well; this is important. I have a feeling that the patient would be motivated to come to the next session after this meeting. In the future, it is worth focusing on exploring the positive resources and well-being through paraphrases, reflections, clarifications (without grades), as well as following the structure of the interview in a way that recognizes the patient’s willingness to provide a lot of different information (e.g., through mini-contracts). Now, finally, do you have any questions for me, or would you like to comment?

## Appendix D

**Criteria for competent judge in SP and VP training in the analysis of diagnostic skills**

The scheme describes what is included in a given SP and VP profile in the dimensions assessed by the diagnostician.

**Profile 1, Miss X, 33**

(1) Positive aspects of functioning:
  - professional area,
  - a sense of belonging,
  - a sense of aesthetics and pleasure from creativity,
  - the ability to present herself in a positive way to others—which benefits her with good emotions,
  - positive affect at parties—relaxed and having fun,
  - a sense of being efficient,
  - intelligence.
(2) Negative aspects of functioning:
  - hysterical;
  - serious difficulties in mentalisation processes;
  - avoidance (avoiding closeness), strong dissociation of unwanted feelings;
  - elements of personality disorders;
  - enters into relationships focused on the exploitation of partners;
  - difficulties in adequately setting short-term and long-term goals;
  - difficulties in maintaining a sense of continuity in life;
  - lack of awareness of the emotional impact she has on others;
  - low level of empathy, shallow relationships, low intimacy;
  - fear of rejection (dependence as dangerous; or ambivalence).
(3) Reactance level—high (high resistance)
(4) Style of coping with stress—externalisation
(5) The stage of readiness for change—pre-contemplation

**Profile 2, Miss Y, 26**

(1) Positive aspects of functioning:
  - Harold the cat—patient learns to contemplate, experience closeness;
  - she likes contact with nature, which improves her mood;
  - a possible experience of feeling close to a partner, caring about a relationship (without sex);
  - a sense of belonging to a group;
  - intelligence, awarded in college;
  - earns her own studies—elements of self-orientation.
(2) Negative aspects of functioning:
  - possibly personality disorder,
  - avoiding attachment style,
  - a negative way of experiencing the world and herself,
  - fear of many areas of life—focused on threat detection—avoidance that impairs functioning,
  - a potentially traumatic experience of abuse,
  - does not set limits—trouble with assertiveness,
  - model victim of violence,
  - high level of suffering,
  - patient for insight therapy.
(3) Reactance level—low (low resistance)
(4) Style of coping with stress—avoidance, internalisation
(5) Readiness for change stage—readiness for change

## Appendix E

**Criteria for competent judges to assess diagnostic rapport written by diagnostician after SP and VP training**

A. ASSESSMENT OF PATIENT’S FUNCTIONING HYPOTHESES

Level of use of available data:

1. Diagnostician focuses on a selected, narrow range of information about the patient;
  2. Diagnostician takes into account a wider range of information;
  3. Diagnostician takes into account the complete set of information provided.

Reference of constructed hypotheses to psychological mechanisms (conceptualization offering an explanation of the patient’s functioning):

1. Diagnostician does not refer to psychological mechanisms, only diagnostic labels (e.g., depressive patient);
  2. Diagnostician refers to diagnostic labels and formulates preliminary conclusions toward explaining a patient’s functioning mechanisms;
  3. Diagnostician formulates hypotheses explaining the mechanisms of the patient’s functioning.

Adequacy of formulated hypotheses in relation to the data

1. Hypotheses formulated in a manner that is inadequate for the material provided;
  2. The hypotheses formulated in relation to the provided data, but omitting a significant part of the material (e.g., information contradicting the formulated hypotheses) or some hypotheses, are adequate—and some are not;
  3. Hypotheses formulated adequately to the provided material.

The occurrence of cognitive errors

(a). Occurrence of confirmation bias:

0—no; 1—yes

(b). Occurrence of multiple alternatives bias:

0—no; 1—yes

(c). Occurrence of the overconfidence bias (the person does not formulate hypotheses in a conditional but a predictive mode and at the same time indicates a high level of confidence in a given hypothesis):

0—no;

1—yes (the person decides a wider category of phenomena, e.g., ‘The patient has a personality disorder’, and indicates the level of confidence in the hypothesis equal to 10);

2—yes (the person decides a narrower category of phenomena, e.g., ‘The patient has borderline personality disorder’, and indicates a level of confidence in the hypothesis equal to 9 or 10).

(d). Occurrence of overconfidence bias (bias level—determined if diagnostician received more than 0 points in the previous variable):

1—The bias is related to one hypothesis;

2—The bias occurs with respect to some of the hypotheses;

3—The bias applies to all hypotheses.

(e). Occurrence of overpathologisation bias (diagnostician focuses only on indicators of psychopathology and describes the occurrence of mental health disorders):

0—no; 1—yes

B. ASSESSMENT OF THE NEGATIVE ASPECTS OF THE PATIENT’S FUNCTIONING (PSYCHOPATHOLOGY)

Adequacy of the assessment in relation to the data

0—Assessment formulated in a manner that is inadequate to the material provided

1—Assessment formulated adequately to the material provided

C. ASSESSMENT OF THE POSITIVE ASPECTS OF THE PATIENT’S FUNCTIONING (PSYCHOPATHOLOGY)

Adequacy of the assessment in relation to the data

0—Assessment formulated in a manner that is inadequate for the material provided

1—Assessment formulated adequately for the material provided

D. EVALUATION OF NOSOLOGICAL DIAGNOSIS HYPOTHESES

Adequacy of formulated hypotheses in relation to the data

1—Hypotheses formulated in a manner that is inadequate to the material provided

2—Most of the hypotheses are formulated in a manner that is inadequate for the provided material

3—Hypotheses formulated in relation to the provided data, but

(a). omitting a significant part of the material or
  (b). the selected hypotheses are not adequate for the provided material

4—Hypotheses formulated adequately for the provided material

## Appendix F

**Example of feedback during the VP training**

(after each decision, the VP program gives a hint of what to do)

**D—diagnostician**

**P—patient**

**D**—What brings you here?

**P**—I’m in a relationship now and it’s an important relationship for me. I hope it will last, I don’t want to lose it.

**VP’s feedback**—The thing is, I can’t have sex and I’m afraid that will be the reason for the break-up, that my boyfriend will get fed up.

1.

**D**—I understand that you feel anxious that your boyfriend could leave you because you are not allowed to have sex. Is there anything in his behaviour to suggest that he might do it?

**P**—I don’t think so, I haven’t noticed any disturbing signals. Jacek is very caring, he keeps telling me about his commitment. I am somehow afraid that I am not giving him what he probably needs in our relationship. After all, every normal person in a partner relationship also expects physical closeness, right?

**VP’s feedback**—Correct paraphrase. Continuation deviates from the patient’s perspective, it is probing ‘as is’. The diagnostician’s task is not to establish the facts, but to build contact and follow the interview structure focused on how the patient experiences the problem.

2.

**D**—You said how supportive your partner behaves towards you. You suppose that he feels uncomfortable in your relationship and that will probably be the reason for the break-up, right?

**P**—Well, actually, maybe it’s not very rational… I don’t know why I do, maybe it’s because of my past experiences. It used to be that I thought it was okay, and then I burnt a lot…

**VP’s feedback**—The diagnostic option selected by the diagnostician aptly refers to the words of the patients. At the same time, it is following the patient’s experience. Indeed—showing her ‘wrong thinking’. The beginning of the interview is not the time for a confrontation.

3.

**D**—Aha, so past experiences have a very strong impact on how you perceive your partner’s behaviour now, right? Could you tell me more about these difficult past experiences?

**P**—Well, I probably could… I just don’t know exactly what to focus on, where to start. I guess it would be easier for me to answer a more specific question—what exactly would I say? This past relationship is actually quite a long time ago.

**VP’s feedback**—A good paraphrase at the beginning. Next, the question about past experiences, deviating from the current problem leading the patient to a diagnostician. This may weaken her motivation to enter into a diagnostic relationship (the patient is motivated to talk about her current difficulty).

4.

**D**—Okay, of course—I will try to formulate the question more specifically. You said that past experiences can influence how you feel about your current relationship. What, in your opinion, could this impact be?

**P**—I don’t know—as I said, this relationship was not a good experience for me. I did not receive negative signals, and later it turned out that I was being cheated on. So now, the fact that there aren’t any signs in Jacek’s behaviour that would prove that he is disappointed doesn’t mean to me that he feels good with me and would not prefer to be with someone who would give him more than emotional closeness, but also physical closeness. Now it also comes to my mind that I was engaged to this ex-partner of mine and not only was there no disturbing or negative signals from him– on the contrary, it was positive—but that he wanted to start a family with me. And then it turned out to be fiction.

**VP’s feedback**—Good intention to relate the past to the actual functioning of the relationship. However, the question is on an abstractive level (‘What is the influence?’), which distances you from talking about the patient’s experience. It is worth exploring further questions about the patient’s experience.

5.

**D**—Let’s go back to your current relationship. Could you give an example of a specific experience in which your anxiety about the relationship was particularly intense—for example when your partner was talking or doing something positive, and you had the impression that it might be such fiction?

**P**—I don’t know, actually. I don’t have such feelings in front of Jacek that he is being dishonest with me. When he says something nice, I know that’s what he means. Rather, I fear that he is not completely happy with me—that this relationship is not what he needs, just because of this lack of sex, because of my blockage. It may be that he is not fully aware of the importance of what I cannot give him.

**VP’s feedback**—The idea of asking for an example of a specific situation where the problem was particularly severe is very good. There was no contact-building intervention—just the introduction of this question. On the other hand, closing the response option with the suggestion of a specific example was unfortunate; the given example could differ from the patient’s experience.

6.

**D**—This is how you guess Jacek’s feelings. You predict that somewhere deep down in his soul he is disappointed, although he may not admit it to himself, right? As if you could understand it better than he did himself. And when you have such an especially clear impression of this—can you recall an example of a specific situation?

**P**—I don’t know, I can’t think of anything now…

**VP’s feedback**—A valuable attempt to paraphrase information about the patient’s feelings in the report. At the same time, in this attempt to paraphrase more strongly than in the patient’s statement, it resounds that the patient ‘guesses’ the partner’s feelings. This statement may be understood by the patient as an objection—that she ‘knows better’ what her partner feels. In this context, asking about an example of a situation may cause the patient’s confusion.

7.

**D**—I see it is difficult to cite an example of such a situation now. If later, during the conversation, something comes to you, please tell me about it.—can we arrange that?

**K**—Well, I think so…

**VP’s feedback**—The diagnostician is rightly not pushing to generate an example of a situation. Unfortunately, the diagnostician does not take responsibility for disrupting the alliance with the patient. The diagnostician places the cause of confusion or the inability to give an example of a situation as being the patient and not the diagnostician’s way of conducting the conversation.

8.

**D**—Let’s focus now on whether you have tried to deal with this problem of a lack of trust in a relationship so far? If so, what have you been doing so far and with what result?

**P**—I am not sure if this is a problem of trust in a relationship… Well, maybe in a way it is, but it seems to me that it is primarily a problem of my block, which could probably destroy any relationship, even the best started—because how much time can one wait for intimacy and sex in a relationship? My boyfriend has been waiting for a long time. I trust that he wants to start a family with me, but with my problem, it does not seem possible, although I would also like it very much.

**VP’s feedback**—A legitimate question about coping methods; however, this will be most effective when the question about coping strategies comes after discussing the essence of the difficulties. Moreover, the term ‘problem of a lack of trust in a relationship’ is far from the way the patient describes her problem; the use of this phrase may disrupt the alliance (e.g., the patient may feel unheard, misunderstood, or judged).

9.

**D**—So, I understand that you say that this is not a problem of trust in the relationship. Could you clarify what the main problem is that brings you here? I am asking this because the very matters related to sex—for example, the inability to have intercourse—are the area of competence of a sexologist rather than a psychologist. I suggest that we focus on the mental aspect of your difficulties—what kind of suffering are you experiencing?

**P**—Well, it probably isn’t the case that I would like to turn to a sexologist (pause)… I know that this is a psychological problem, this blockage I have. I have been to the gynaecologist and I know there are no physical reasons for this problem…

**VP’s feedback**—The diagnostician’s intervention provokes the patient to explain why she does not seek help elsewhere. The patient may perceive this as questioning her (pro-health) decision to seek psychological help. An additional problem is the use of jargon (‘mental aspect of difficulties’) and the request that the patient adjust the description of her experience to the jargon phrases used.

10.

**D**—Of course, you can seek psychological help and you can get it here. My point is to clarify your expectations—a sexologist is a person with different competencies than a gynaecologist and deals not only with the physiological side of functioning but with human sexuality in all its complexity, including the psychological elements of experiencing intercourse.

**P**—I don’t know, I didn’t think about it that way. I thought I should ask a psychologist and my boyfriend thinks so too because I have other difficulties, not only those related to sex. My boyfriend insisted that I come here because we’ve been talking mainly about these other issues lately, in my job…

**VP’s feedback**—The diagnostician attempts to use psychoeducation. It may be a valuable tool but only in the context of the patient’s explicit order for this type of intervention (the patient asks for this kind of help or confirms that expectation). Offering psychoeducation ‘out-of-the-box’ might be perceived as lecturing, and this can disrupt contact.

11.

**D**—You say that the initiative to come here came from your boyfriend, right? I wonder if you would have decided to come to today’s meeting without his insistence. Can we talk for a moment about how you feel about the idea of using a psychologist?

**P**—I’m actually glad my boyfriend persuaded me to come here. I would probably not have made up my own mind, but that was for financial reasons because, apart from that, I hoped that talking to a psychologist would be helpful…

**VP’s feedback**—It is valuable to ask about the patient’s motivation. However, the form of the question (‘would you have decided (…) without his insistence?’) questions the patient’s sense of agency—and this is not pro-health. Additionally, the diagnostician moves away from the threads introduced by the patient as those relating to her difficulties and which she would like to deal with.

12.

**D**—I hear that you needed psychological help. It is good that you decided to ask for it. At the same time, you spoke about your problems at work and financial difficulties. Can I ask what exactly these problems concern in your work?

**P**—Yes. I have a job that was supposed to be undemanding, and the opposite is true. I work in a shopping centre at a clothing store. I have a very rude boss. She is very nervous, you could say that she takes things out on us—not only on me but on all the girls who work for her. She pays us little, and sometimes our pay is transferred late. And she gives me the worst jobs to do, although at the beginning we agreed that it would be different.

**VP’s feedback**—Reflecting the patient’s need to use help is positive. The return to the threads reported by her as problematic (work, finances) is also an important signal of careful listening. It is worth remembering, however, that these difficulties were not reported by the patient as the most important. It is important during the first consultation to focus first on the problem identified by the patient as the most troublesome and then on other difficulties reported by the patient.

13.

**D**—I hear that contact without is very difficult for you. She is unpleasant to all employees, creates a very bad atmosphere, which makes your work a heavy burden for you. I think it is worth returning to this topic later, but now I propose to focus on what you spoke about in the first place—the inability to have sex. Since when have you been experiencing this blockage?

**P**—[pause] Actually, since my first relationship, you could say forever… This is the kind of trauma I have had since my teenage years.

**VP’s feedback**—The diagnostician rightly directs the attention to the issue that the patient raised in the first place, without, however, allowing the patient to co-decide on the direction of the conversation. If the patient had a choice as to whether she would like to continue talking about the topic of work or return to the issue of the sexual block, it would be an invitation to a partner relationship, and therefore, it would build good contact.

14.

**D**—You talk about a very difficult topic, which is also very important for understanding the nature of your problem. I realize that talking about the trauma you experienced as a teenager may be a challenge in our first meeting. Your safety in conversation on this topic is key to me, so please pay attention to your well-being at all times, okay? Please tell me about this trauma, so that you can understand what brings you here and be able to plan your therapy accordingly. Now, please tell me about what happened, what was this trauma?

**P**—I understand that it is important to understand my difficulty… So maybe I will try, but I’m not feeling very well… Could I open a window here?

**VP’s feedback**—It is good that the diagnostician recognizes the importance of the patient’s difficulties and realizes that talking about her injury may be very threatening for her, especially during the first diagnostic meeting. For this reason, the diagnostician should not encourage her to reveal the details of the traumatic experience, nor expect the patient to monitor her well-being and signal negative changes in this regard. It is breaking borders and risking retraumatisation.

15.

**D**—Your emotional arousal shows that you are very stressed. Some people react to severe stress with somatic symptoms. Of course, we can open the window [gets up and opens the window]. Can we stop this tension for a moment? If you were to rate it on a scale from zero, where zero is complete lack of tension, complete relaxation, to one hundred—where one hundred is the strongest stress you have experienced in your life, then where would you place your current state of mind and level of tension?

**P**—It’s hard to say… Zero is complete relaxation and a hundred is the strongest stress, right? It’s probably somewhere around fifty-sixty now…

**VP’s feedback**—A positive attempt to distance the patient from the experienced tension using the scaling technique. The beginning of the intervention, however, is a suggestion not agreed with the patient (that the patient is somatising). Additionally, the diagnostician did not withdraw directly from the announcement of further discussing the traumatic experiences, which may maintain/increase the patient’s tension.

16.

**D**—I understand this is a high level of arousal. So we don’t need to go further on this topic, which has such tense memories. The fact that they are so difficult is very relevant and sufficient information for me. Do you feel ready to return to our conversation, to the topics previously discussed by you?

**P**—Yes, I think so. So what exactly should we go back to?

17.

**D**—We started our conversation with the topic of the blockade in the sexual sphere. Later, you also talked about the difficulties in your work. Would you like to focus on any of these topics now, or is there anything else you would like to talk about?

**P**—Maybe at work. I know it’s such a bad place for me. I wanted to work there so that it would be easy for me to combine earnings with studies, but the choice of the workplace turned out to be not the best. Maybe, I should have looked for something else right away, I don’t know…

18.

**D**—I understand that you feel bad at work.

**P**—Yes, very much. But I’m not the only one—all the girls are afraid of the boss, most of them leave quickly, the staff turnover is very high. And somehow I still hang on in there, although when I think about having to go there in the morning, I feel like crying.
